# Eribulin for Treating Locally Advanced or Metastatic Breast Cancer After One Chemotherapy Regimen: An Evidence Review Group Perspective of a NICE Single Technology Appraisal

**DOI:** 10.1007/s41669-018-0114-z

**Published:** 2019-02-11

**Authors:** Nigel Fleeman, Adrian Bagust, Rui Duarte, Marty Richardson, Sarah Nevitt, Angela Boland, Eleanor Kotas, Joanne McEntee, Nicky Thorp

**Affiliations:** 1 Liverpool Reviews and Implementation Group, University of Liverpool, Whelan Building, Liverpool, L69 3GB UK; 2 North West Medicines Information Centre, Liverpool, L69 3GF UK; 3 The Clatterbridge Cancer Centre NHS Foundation Trust, Bebington, Wirral CH63 4JY UK

## Abstract

Eribulin is a recommended treatment option for locally advanced or metastatic breast cancer (LABC/MBC) in adults whose disease has progressed after at least two chemotherapy regimens. The National Institute for Health and Care Excellence (NICE) invited the manufacturer of eribulin (Halaven^®^; Eisai Ltd) to submit evidence for the clinical and cost effectiveness of eribulin for treating LABC/MBC after one chemotherapy regimen in accordance with the institute’s Single Technology Appraisal (STA) process. This article presents a summary of the company’s evidence, Evidence Review Group (ERG) review and resulting NICE guidance (TA515), issued 28 March 2018. Clinical evidence for eribulin versus capecitabine in LABC/MBC was derived from a subgroup of 392 patients with human epidermal growth factor receptor (HER2)-negative disease which had progressed after only one prior chemotherapy regimen for LABC/MBC in the phase III, randomised, controlled Study 301 (*n* = 1102). Overall survival (OS) but not progression-free survival (PFS) was improved for patients treated with eribulin versus capecitabine in this subgroup. Using the discounted patient access scheme price for eribulin, the company developed a de novo economic model. In the base case, the incremental cost-effectiveness ratio (ICER) for eribulin versus capecitabine was £36,244 per quality-adjusted life year (QALY) gained. However, the ERG identified several problematic issues relating to modelling OS and PFS, drug costing and utility values, and made ten revisions to the company model. The overall impact of all ten revisions was to increase the ICER per QALY gained by £46,499. The Appraisal Committee (AC) accepted all changes made by the ERG except for the change to utility values; the AC considered that the value should be mid-way between the company’s and the ERG’s preferred values. A modified model was submitted by the company that included this utility value, but maintained some elements of the base case that the AC had been critical of (differential PFS between treatment arms and application of treatment cap). The new model also included a ‘blended’ comparator (capecitabine and vinorelbine). The AC noted there was no evidence to support a ‘blended’ comparator, differential PFS between treatment arms or a treatment cap. The AC therefore concluded that the most plausible ICER was likely to be £69,843 per QALY gained (derived from an ERG sensitivity analysis using the AC’s preferred utility value, no differential PFS and no treatment cap). Therefore, eribulin was not recommended for treating LABC/MBC in adults who have had only one chemotherapy regimen.

## Key Points for Decision Makers

**Table:** 

Clinical effectiveness evidence was presented for a subgroup of patients with human epidermal growth factor receptor (HER2)-negative disease that has progressed after only one prior chemotherapy regimen for locally advanced or metastatic breast cancer (LABC/MBC). The evidence showed an improvement in overall survival (OS) but not in progression-free survival (PFS) for patients treated with eribulin versus those treated with capecitabine in this subgroup.
In contrast to the clinical effectiveness evidence, the cost-effectiveness analysis submitted by the company assumed a small PFS benefit for eribulin versus capecitabine (and versus capecitabine or vinorelbine) and a maximum duration of treatment (in both treatment arms).
In 2018, the National Institute for Health and Care Excellence (NICE) Appraisal Committee (AC) rejected these assumptions. The NICE AC did not recommend eribulin for treating LABC/MBC in adults who have had only one chemotherapy regimen.
Previously, in 2016, NICE had recommended eribulin for LABC/MBC that has progressed after at least two chemotherapy regimens. Eribulin is still recommended for treating LABC/MBC in adults whose disease has progressed after at least two chemotherapy regimens.

## Introduction

The National Institute for Health and Care Excellence (NICE) is an independent organisation responsible for providing national guidance to the National Health Service (NHS) in England and Wales on a range of clinical and public health issues, including the appraisal of new health technologies. The NICE Single Technology Appraisal (STA) process is specifically designed for the appraisal of a single health technology for a single indication, where most of the relevant evidence lies with one manufacturer or sponsor and typically covers new technologies shortly after UK market authorisation is granted [1]. Within the STA process, the manufacturer or sponsor provides a written submission (alongside a decision-analytic model) that summarises the estimate of the clinical effectiveness and cost effectiveness of the technology. An external independent organisation (typically, an academic group) known as the Evidence Review Group (ERG) provides a critique of the company’s submission (the ERG report). Consultees, clinical specialists and patient representatives also provide additional information during the appraisal process.

Using a specification developed by NICE (the final scope), the NICE Appraisal Committee (AC) considers the company’s submission, the ERG report and testimonies from experts and stakeholders in order to determine whether the technology represents clinically effective and cost-effective use of NHS resources. All stakeholders and the public have an opportunity to comment on the preliminary guidance issued by NICE in the form of an Appraisal Consultation Document (ACD), after which the AC meets again to produce the final guidance (Final Appraisal Determination [FAD]). The final guidance constitutes a legal obligation for NHS providers in England and Wales to provide a technology that is approved within its licensed indication [1].

This article presents a summary of the ERG report for the STA of eribulin for treating locally advanced or metastatic breast cancer (LABC/MBC) after one chemotherapy regimen. The Liverpool Reviews and Implementation Group at the University of Liverpool was commissioned to act as the ERG for this STA. Full details of all relevant appraisal documents (including the appraisal scope, ERG report, company and consultee submissions, NICE guidance and comments on each of these) can be found on the NICE website [2].

## The Decision Problem

Breast cancer is the most common cancer in the UK, accounting for almost a sixth (15%) of all new cancer cases [3]. It has been reported that as many as 35% of women diagnosed with early breast cancer will eventually develop LABC/MBC and approximately 85% of patients with LABC/MBC have human epidermal growth factor receptor (HER2)-negative disease [4]. Symptoms experienced by patients with LABC/MBC can be severe [5]. There is currently no cure for LABC/MBC, and the long-term prognosis is poor [4].

Eribulin is a first-in-class anti-neoplastic agent [4], administered intravenously over 2–5 min on days 1 and 8 of every 21-day cycle [6]. It was originally licensed in 2011 by the European Medicines Agency (EMA) for the treatment of adult patients with LABC/MBC who had progressed after at least two chemotherapy regimens for advanced disease [7]. In guidance issued by NICE in 2012, eribulin was not recommended as a routine treatment option for these patients in England and Wales [8, 9].

In 2014, the EMA granted an extension to the 2011 indication for eribulin enabling eribulin to be used earlier in the treatment pathway [7]. Eribulin is now indicated for the treatment of adult patients with LABC/MBC who have progressed after at least one chemotherapeutic regimen for advanced disease. Prior therapy should have included an anthracycline and a taxane in either the adjuvant or metastatic setting unless patients were not suitable for these treatments [6].

In 2016, NICE recommended eribulin as an option for treating LABC/MBC in adults when the disease has progressed after at least two chemotherapy regimens (which may include an anthracycline or a taxane, and capecitabine), but only if the company provides eribulin with the discount agreed in the patient access scheme (PAS) [10]. Also in 2016, NICE issued an updated scope for the appraisal of eribulin for treating LABC/MBC after one chemotherapy regimen (interpreted by the company and ERG as meaning after only one prior chemotherapy regimen) [11]. In 2017, NICE requested that the ERG examine the evidence for the population identified in the new scope (i.e. treatment after one chemotherapy regimen).

The focus of the population in the company submission (CS) was narrower than the population described in the updated scope issued by NICE [11] as it focussed only on patients with HER2-negative LABC/MBC (whose disease had progressed after only one prior chemotherapy regimen in the advanced setting) [4]. This population is hereafter referred to as subgroup 1. The comparators were capecitabine or vinorelbine. Both of these chemotherapy drugs are currently recommended as second-line treatment options by NICE for patients with LABC/MBC [5].

## Independent Evidence Review Group Review

The evidence provided by the company comprised an initial submission [4], an economic model and the company’s response to the ERG’s clarification requests [4]. The ERG report comprised a summary and critical review of the evidence for the clinical and cost effectiveness of the technology provided by the company [4]. The role of the ERG was to:

- Assess whether the company’s submitted evidence conforms to the methodological guidelines issued by NICE [12].
- Assess whether the company’s interpretation and analyses of the evidence are appropriate.
- Indicate the presence of other sources of evidence or alternative interpretations of the evidence that could help inform the development of NICE guidance.

In addition to providing this detailed critique, the ERG updated the company searches and modified a number of key assumptions and parameters within the company’s economic model to examine the impact of these changes on cost-effectiveness results. The ERG also critically reviewed evidence provided after publication of the ACD [13].

### Clinical Evidence

Clinical effectiveness evidence was derived primarily from Study 301, a multi-centre, phase III, open-label, randomised controlled trial comparing eribulin with capecitabine as first-, second-, or third-line therapy for the treatment of LABC/MBC [14]. Only data for patients who have received only one prior chemotherapy regimen for LABC/MBC (i.e. second-line therapy) were directly relevant to this appraisal.

A total of 1102 participants were randomised in Study 301; 554 to the eribulin treatment arm and 548 to the capecitabine treatment arm [14]. A total of 392 participants (35.6%) randomised in Study 301 were included in the subgroup 1 population (HER2-negative disease that has progressed after only one prior chemotherapy regimen): 186 in the eribulin treatment arm and 206 in the capecitabine treatment arm. Patient characteristics were well balanced across treatment arms [4].

In the overall trial population of Study 301, there was no statistically significant difference in terms of overall survival (OS) between patients treated with eribulin and patients treated with capecitabine (15.9 months vs 14.5 months; hazard ratio [HR] = 0.879, 95% confidence interval [CI] 0.77–1.00) [14]. However, in the post hoc analysis of the subgroup 1 population, OS was statistically significantly improved for patients treated with eribulin compared with capecitabine (16.1 months vs 13.5 months; HR = 0.77, *p* = 0.026) [4]. No statistically significant differences were observed for progression-free survival (PFS) in the overall trial population or the subgroup 1 trial population. Median PFS was approximately 4 months in both treatment arms of the trial (overall trial population and subgroup 1) [4, 14].

Most adverse event (AE) data presented by the company were only presented for the overall trial population of Study 301 [4]. These data show that most patients in both treatment arms experienced an AE (94.1% with eribulin, 90.5% with capecitabine). Most AEs were considered treatment related in both treatment arms (84.6% with eribulin, 77.1% with capecitabine). There were few differences between treatment arms in terms of AEs that led to dose delays (31.8% with eribulin, 35.7% with capecitabine) or dose reductions (32.0% with eribulin, 31.9% with capecitabine). Fatal AEs were reported by 4.8% of patients treated with eribulin and 6.6% of patients treated with capecitabine. In the subgroup 1 population, neutropenia (53.3% vs 14.6%), leucopenia (31.0% vs 9.3%), pyrexia (14.1% vs 4.9%), peripheral sensory neuropathy (16.3% vs 4.9%) and alopecia (34.8% vs 2.9%) were all much more common with eribulin than capecitabine [4]. In contrast, the incidences of diarrhoea (14.1% vs 24.9%) and palmar-plantar erythrodysaesthesia syndrome were much lower (0.5% vs 48.3%) with eribulin than capecitabine. Other AEs reported by ≥ 20% of patients in either treatment arm of the subgroup 1 population included asthenia/fatigue (31.5% vs 25.4%), anaemia (21.2% vs 19.5%) and nausea (20.7% vs 21.0%). The frequencies of the AEs cited for either treatment arm in the subgroup 1 population were similar to the frequencies reported for the overall trial population [4, 14].

Results from health-related quality of life (HRQoL) analyses were not presented for the subgroup 1 population, but instead were presented for the overall trial population (*n* = 1062 at baseline) and/or for all patients with HER2-negative disease (*n* = 718 at baseline) [4]. Overall, the median Global Health Status (GHS)/quality-of-life (QoL) scores measured using the European Organisation for Research and Treatment of Cancer Quality of Life Questionnaire C30 (version 3.0) (EORTC QLQ-C30) [15] in the overall trial population were similar in the eribulin and capecitabine arms. The majority of patients (≥ 74%) in both treatment arms maintained or improved their GHS/QoL scores versus their baseline scores at 6 weeks, 3 months and 6 months. Similar findings were observed in all patients with HER2-negative disease. The results of the other HRQoL analyses reported in the CS are based on post hoc analyses of Study 301 data [4]. These findings suggested diminished HRQoL for patients treated with eribulin for systemic therapy side effects (dry mouth, distorted sense of taste, conjunctivitis, hair loss, feeling ill/unwell, hot flushes, and headaches) and for patients treated with capecitabine for gastrointestinal side effects (nausea, vomiting and diarrhoea). Patients receiving eribulin had comparatively worse scores than patients receiving capecitabine for body image and sexual functioning as measured by the Quality of Life Questionnaire BR23 (version 1.0) (QLQ-BR23) [16]. On the other hand, a higher proportion of patients receiving capecitabine reported a meaningful worsening on the ‘future perspective’ scale than those receiving eribulin.

### Critique of the Clinical Evidence and Interpretation

Overall, the ERG was satisfied with the clinical effectiveness systematic review process as described in the CS. However, the ERG’s updated literature searches identified subgroup analyses of data from Study 301 that were published after the company had completed its searches [17]. The results from this paper show that there is no statistically significant difference in OS between treatment arms in the licensed population (*n* = 882), i.e. patients with one or more prior chemotherapy regimens regardless of HER2 status (HR = 0.87, 95% CI 0.75–1.01, *p* = 0.06). However, there was a statistically significant gain in OS for all HER2-negative patients enrolled into the trial (*n* = 755) treated with eribulin compared to those treated with capecitabine (HR = 0.84, 95% CI 0.71–0.98, *p* = 0.03) [17]. For the subgroup of patients with HER2-negative status who have also had at least one prior chemotherapy regimen for LABC/MBC (*n* = 595), there was a trend towards an OS gain for eribulin versus capecitabine (HR = 0.84, 95% CI 0.70–1.00, *p* = 0.05) [17]. In response to a clarification query from the ERG, the company reported a similar result for patients who had received only one prior chemotherapy regimen for advanced disease (*n* = 573), regardless of HER2 status (HR = 0.83; 95% CI 0.69–1.00, *p* = 0.05) [4]. There were no statistically significant differences in PFS for any of these subgroups [4, 14]. Overall, therefore, the findings suggest that patients with HER2-negative disease treated with eribulin have improved OS when compared with patients treated with capecitabine.

The ERG considered that AE data from Study 301 do not suggest that there are any safety concerns from treatment with either eribulin or capecitabine [4, 14]. Due to diminishing sample sizes over time (questionnaires were completed until disease progression or initiation of other antitumor treatment), the ERG considered that the HRQoL data from Study 301 [4] should be treated with caution.

While no evidence was presented comparing eribulin with vinorelbine [4], the ERG noted that in a previous appraisal of eribulin (TA423), the clinical expert present at the AC meeting stated that most patients in the NHS receive capecitabine as a second-line treatment for LABC/MBC [10]. Capecitabine is arguably, therefore, the most appropriate comparator for patients who have received only one prior chemotherapy regimen for LABC/MBC.

Based on the baseline data presented [4, 14], the ERG considered that the patient population in Study 301 appeared to be younger than patients seen in clinical practice in England. In addition, the ERG noted that only a minority of patients were from Western Europe, with no patients recruited from the UK. Nonetheless, noting other trial and patient baseline characteristics, the ERG considers the results of the trial likely to be generalisable to clinical practice in England.

### Cost-Effectiveness Evidence

The company carried out a literature review to identify relevant cost-effectiveness studies, but none were identified [4]. The company, therefore, developed a de novo economic model to compare the cost effectiveness of treatment with eribulin versus capecitabine for the subgroup 1 population [4]. The model comprised three mutually exclusive health states: pre-progression or stable disease, post-progression or progressive disease, and dead. All patients entered the model in the stable health state and remained in this state until disease progression. In the base case, the model time horizon was set at 5 years with monthly cycles. The model perspective was that of the UK NHS. Outcomes were measured in quality-adjusted life years (QALYs), and both costs and QALYs were discounted at an annual rate of 3.5%, as recommended by NICE [1]. The following costs were included in the model: costs of the intervention and comparator drugs, administration costs, treatment-related monitoring costs, costs for treating AEs, post-progression treatment costs, and palliative and terminal care costs. Costs were valued at 2014–2015 prices.

Survival was estimated based on data from Study 301 [4]. Utility values were mapped to EuroQol-5 dimension (EQ-5D) values from the responses of patients in Study 301 who completed the EORTC QLQ-C30 questionnaire [4]. Resource use and costs were estimated based on information from Study 301 [4], published sources [18, 19] and clinical experts. As a PAS offering a discount to the list price of eribulin had been formally agreed between the company and the Department of Health in 2016, this price was used in the company’s cost-effectiveness analysis.

In the base case, eribulin generated greater benefit than capecitabine, but at increased cost. The company base case incremental cost-effectiveness ratio (ICER) for eribulin versus capecitabine was estimated to be £36,244 per QALY gained [4].

The company carried out a range of deterministic sensitivity analyses. The resultant ICERs ranged from £32,095 to £47,148 per QALY gained [4], i.e. ranging from £4149 less than the base case to £10,904 greater than the base case.

The company’s probabilistic sensitivity analysis (PSA) involved varying only a limited number of parameters. There was a 20% probability of treatment with eribulin being cost effective at a threshold of £30,000 per QALY gained and a 69% probability of eribulin being cost effective at a threshold of £50,000 per QALY gained [4].

The company carried out six scenario analyses. Increasing the time horizon to 20 years had the largest impact and lowered the ICER to £29,743 per QALY gained (an 18% reduction from the base case result) [4].

### Critique of the Cost-Effectiveness Evidence and Interpretation

The company used Kaplan–Meier (K–M) data from Study 301 directly to model OS in the base case analysis. The company appended projective functions to the K–M data from 5 years onwards to model OS in the scenario analyses where the time horizons were varied. The ERG’s analysis showed that the method by which the company appended projections to the K–M data yielded an underestimate of OS gain for treatment with eribulin. This underestimation had a small effect on the 5-year base case results, but was more pronounced in results of the time horizon scenario analyses.

The company also modelled PFS using the K-M data from Study 301 for each treatment arm separately. This yielded a mean progression-free benefit of 0.57 months in the base case. However, examination of PFS trial data by the ERG suggested a close correspondence between the timing of progressive disease developing regardless of the treatment used. To test this hypothesis, the ERG re-ran the K–M analysis. This showed that there is no statistically significant difference between the risks of suffering disease progression in the two treatment arms (log-rank test *p* = 0.131, Breslow test *p* = 0.071, and Tarone-Ware test *p* = 0.106), reflecting the clinical effectiveness evidence, which showed no statistically significant difference between treatment arms.

The ERG identified several issues relating to the way in which the company costed drugs. First, two logic errors were identified, one relating to the cost of administering eribulin and the other concerning the cost of oral vinorelbine used after progression. Second, the ERG identified errors linked to the body surface area (BSA) values used to calculate the acquisition costs of eribulin and subsequent chemotherapy, including drug wastage. Third, a dose intensity multiplier that only had an effect when the company’s alternative approach to calculating drug costs (i.e. excluding wastage) was applied. Fourth, the company assumed that treatment continued throughout the period from randomisation to disease progression, disregarding a proportion of patients who discontinued treatment before progression. Fifth, the company provided two approaches to estimating the cost of further lines of chemotherapy: limiting the number of cycles of therapy overall (in the base case to no more than eight cycles) and treating until progression, which meant that none of the patients who progressed whilst on eribulin or capecitabine incurred the costs associated with any subsequent chemotherapy (fourth, fifth, etc., lines of treatment). Both of these approaches led to anomalous results. The first option completely ignored an important component of differential costs, that patients who achieved a good response to third-line treatment would, on average, continue third-line therapy for a longer period than those with poor response and may subsequently have a better performance status leading to a greater probability of proceeding to further lines of treatment. The second option effectively capped the cost of all subsequent treatments. This resulted in a bias in favour of eribulin, since the ERG’s analysis of post-progression survival (PPS) data showed that eribulin treatment was associated with additional PPS time and, therefore, led to more use of additional lines of treatment (all of which would result in additional associated costs).

The ERG questioned the appropriateness of applying the algorithm published by Crott and Briggs in 2010 [20] to convert EORTC QLQ-C30 values to EQ-5D utility values. The algorithm was based on data made available from a historical clinical trial, which recruited patients from 1993 to 1996 (median follow-up 5.5 years) and compared two chemotherapy regimens. In stark contrast to Study 301 [14], the published trial results [21] indicated that only untreated patients with locally advanced (but not metastatic) breast cancer and good performance status were recruited, and only neo-adjuvant treatments were administered. Furthermore, the ERG noted that the value used in the company model to represent the HRQoL of patients with stable disease (but not responding to treatment) was very similar to the value for progressed disease (0.70 vs 0.68) and considered this level of similarity to be implausible.

Three further issues were identified by the ERG. First, within the company model, costs and benefits were discounted on a continuous basis rather than annually in line with NHS budgeting and accounting years. Second, the method employed by the company to carry out PSA did not take into account uncertainty related to correlated values; furthermore, drug costs were only varied in a deterministic manner. Third, the ERG did not consider that the company explored parameter uncertainty sufficiently.

Given the above, the ERG made the following modifications to the model:

1. Use of ERG preferred PFS estimates (pooled analysis of PFS data from both treatment arms).
2. Use of ERG preferred OS estimates (examining the trends in cumulative hazard plots of the trial data, identifying the time point in each treatment arm where a long-term exponential trend becomes established and replacing the trial K-M data at the time point at which the calibrated trend lines most closely replicated the trial data).
3. Use of annual rather than continuous discounting.
4. Use of time to treatment discontinuation rather than PFS for costing treatments.
5. Use of the ERG revised unit cost of eribulin (using the distribution of BSA in individual patients using the standard deviation, as opposed to the standard error used by the company).
6. Use of the ERG revised unit costs of other drugs (correction of BSA values as in R5 and excluding BSA values from patients considered to receive adjuvant, neo-adjuvant or palliative care when calculating the mean BSA).
7. Use of ERG alternative utility value for progressed disease using a utility value set published by Lloyd et al. 2006 [22] specifically for breast cancer patients receiving chemotherapy using the standard gamble methodology.
8. Use of ERG method for estimating subsequent therapy costs (removing the treatment cap of 8 months and amending the proportion of breast cancer patients progressing between lines of therapy from first to fifth).
9. Correction of logic error in calculating eribulin administration costs.
10. Correction of logic error in calculating cost of oral vinorelbine.

The revisions with the largest individual impacts on the company’s base case ICER per QALY gained were R1 (+£14,621), R7 (+£10,904) and R8 (+£11,109). Taken together, the overall impact in the base case ICER per QALY gained was to increase the ICER per QALY gained to £82,743 (i.e. an increase of £46,499).

### Conclusions of the ERG Report

The population described in the updated NICE scope (patients with LABC/MBC whose disease has progressed after only one prior chemotherapy regimen in the advanced setting) is a subgroup of the population for whom eribulin is indicated (patients with LABC/MBC whose disease has progressed after at least one prior chemotherapy regimen in the advanced setting). The company only presented evidence for a subgroup of the NICE scope, subgroup 1 from Study 301 defined as patients with HER2-negative LABC/MBC whose disease has progressed after only one prior chemotherapy regimen in the advanced setting [4]. The ERG considers the results from the analyses of this subgroup are likely to be generalisable to clinical practice in England.

Almost fully mature efficacy data from Study 301 do not show any statistically significant differences in OS or PFS between eribulin and capecitabine when the overall trial population (*n* = 1102) with LABC/MBC is treated [14]. Similar results are observed for the 573 patients who have received only one prior chemotherapy regimen for LABC/MBC [4]. Results of the efficacy analyses for the subgroup 1 population of 392 patients with HER2-negative disease, however, show a statistically significant improvement in OS, but not in PFS, for patients treated with eribulin versus capecitabine [4].

The safety profile associated with eribulin differs to that of capecitabine: in Study 301, the incidences of neutropenia, leucopenia, pyrexia, peripheral neuropathy and alopecia were all higher with eribulin than with capecitabine, whereas the incidences of diarrhoea and palmar-plantar erythrodysaesthesia syndrome were lower [4, 14]. Dose intensity was high for both eribulin and capecitabine, suggesting that both drugs appear to have manageable safety profiles [4, 14]. No statistically significant or clinically meaningful difference between eribulin and capecitabine in the pre-specified measure of HRQoL, GHS/QoL, was reported for the overall trial population of Study 301 or for the subgroup of patients with HER2-negative disease [4].

In terms of cost effectiveness, the ERG considers that the company substantially underestimated the size of the most probable base case deterministic ICER per QALY gained for eribulin versus capecitabine in the subgroup 1 population. Using the PAS price for eribulin, the company’s base case ICER is £36,244 per QALY gained, which is £46,499 less than the ICER estimated by the ERG (£82,743 per QALY gained).

The analysis of time-to-event data from Study 301 shows that eribulin provides no additional benefit compared to capecitabine prior to disease progression. However, there is evidence to suggest that there is a modest improvement in survival following disease progression that can be attributed to treatment with eribulin. Further research may be warranted to explore this unusual effect.

## National Institute for Health and Care Excellence Guidance

At the first AC meeting, the AC was minded not to recommend eribulin for treating LABC/MBC in adults who have had only one chemotherapy regimen. Following the company’s response to the ACD and their submission of additional data [13], a second AC meeting was held. NICE guidance was issued on 28 March 2018: eribulin was not recommended for treating LABC/MBC in adults who have had only one chemotherapy regimen [10].

### First Appraisal Committee Meeting

A number of observations were made by the AC at the first meeting from its discussions with the patient and clinical expert and consideration of the evidence presented by the company and the ERG’s critique. The key issues arising are summarised below.

#### Consideration of the Clinical Effectiveness Issues

Following discussion with the clinical expert, the AC concluded that the most relevant comparator for the majority of patients with LABC/MBC who had had only one chemotherapy regimen was capecitabine. While the AC noted that the eribulin marketing authorisation includes people with HER2-positive and HER2-negative disease, the AC accepted that only patients with HER2-negative disease were relevant for the current appraisal. However, the AC did express concerns about the reliability of the results from a post hoc subgroup analysis (subgroup 1) of Study 301 [4]. The AC considered that the greatest uncertainty surrounded the question of why there was an increase in OS but not in PFS for eribulin versus capecitabine in this subgroup. Importantly, it was uncertain if the benefit in OS occurred as a result of treatment with eribulin or as a result of the subsequent treatments received following eribulin. The AC considered, therefore, that the question most clinically relevant to the current appraisal was whether receiving eribulin before capecitabine was more clinically and cost effective than the current practice of having eribulin after capecitabine. The AC concluded that analyses of the available clinical effectiveness data did not address this question.

#### Consideration of the Cost-Effectiveness Issues

The AC considered that the company’s economic model, with the ERG’s error corrections, was suitable for its decision-making. The AC’s considerations focussed on the three model inputs that were key drivers of the cost-effectiveness results. First, the AC explored the modelling of PFS. The AC noted that there was no statistically significant PFS benefit reported in the clinical effectiveness evidence and so preferred the ERG’s approach to modelling (pooled data across treatment arms, therefore, no PFS benefit) to that of the company (a PFS benefit of 0.57 months for eribulin vs capecitabine). Second, the AC considered utility values. The AC concluded that the most plausible utility values were likely to be somewhere between the company’s and ERG’s estimates. Third, the AC heard from the clinical expert that it was realistic to assume that most patients would still be having active treatment more than 8 months after starting eribulin. Therefore, the AC concluded that the 8 month cap on total treatment, as applied by the company, was not clinically plausible. The AC concluded that the most plausible ICER per QALY gained was likely to be closer to the ERG’s estimate than to the company’s base case estimate.

#### Consideration of End-of-Life Criteria

The AC noted the company’s model predicted a mean OS with capecitabine of about 17 months. Evidence from Study 301 showed a mean OS benefit of more than 3 months for eribulin compared with capecitabine in the intention-to-treat (ITT) population of Study 301 [4]. The AC therefore concluded that eribulin met the NICE end-of-life criteria, meaning that eribulin could be considered cost effective at a higher threshold (typically ≤ £50,000 per QALY gained) than normally would be the case (typically ≤ £30,000 per QALY gained).

### New Evidence Considered by the Appraisal Committee

Given the issues discussed and the decision reached at the first AC meeting, the company stated in its response to the ACD that it did not agree that the submitted subgroup evidence was not sufficiently robust for decision-making [13]. The company also stated that it did not agree that the OS benefit in the trial would not be directly attributable to eribulin alone, nor did it agree that the summary of the cost-effectiveness evidence was a reasonable interpretation of the evidence [13]. New data presented by the company and the ERG’s critique of the company’s response are summarised below.

#### Evidence for Clinical Effectiveness

Like the company, the ERG had no major concerns about the robustness of using subgroup data for decision-making [13]. The ERG reiterated its earlier conclusions that the clinical effectiveness evidence suggests that all patients with HER2-negative disease treated with eribulin have improved OS when compared with patients treated with capecitabine [13, 17], as do patients in subgroup 1 [4].

To support its claim that the OS benefit in the trial can be attributed to eribulin alone, the company presented results from exploratory ad hoc analyses [13]. Most of the data were presented for the ITT population, not for subgroup 1. In the ITT population, median OS for patients who received no subsequent treatment was very similar in both treatment arms (eribulin 7.4 months, capecitabine 7.1 months). Also, in the ITT population, median OS for patients who received eribulin followed by capecitabine (18.3 months) was lower than for those who received eribulin followed by any other active treatment (19.9 months), but the same as that for patients who received capecitabine followed by any other active treatment (18.3 months). In the subgroup 1 population, censoring patients if they had crossed over to either eribulin or capecitabine after disease progression showed OS was statistically significantly improved for patients who received eribulin compared to patients who received capecitabine. However, a large proportion of patients treated with eribulin crossed over to receive capecitabine (≥ 50%), but very few patients actually crossed over from capecitabine to eribulin (< 1%). Furthermore, patients in both treatment arms also received other subsequent treatments. Overall, the ERG considered that it was difficult to draw any firm conclusions from the data presented by the company [13].

#### Evidence for Cost Effectiveness

The new cost-effectiveness evidence presented by the company included a utility value midway between their previously preferred value and the ERG value, resulting in an ICER of £69,843 per QALY gained [13]. The company also increased the maximum number of months of treatment (eribulin and subsequent) that patients could receive from 8 months to 21 months, resulting in an ICER of £74,545 per QALY gained [13]. The ERG continued to consider that imposing a cap on the duration of treatment was not justified [13]. The company continued to model a PFS benefit for eribulin versus the comparator resulting in an ICER per QALY gained of £76,838 [13]. The company also changed the comparator arm in its economic model from 100% capecitabine to 50% capecitabine and 50% vinorelbine (all intravenous) resulting in an ICER per QALY gained of £71,649 [13]. The ERG noted that changing the comparator assumed that the two treatments have equal efficacy and an equal safety profile, which was not supported by evidence. Moreover this assumption also altered the balance of treatment-related costs in favour of eribulin [13].

Overall, the ERG did not consider there was good reason to accept any of the model changes proposed by the company. Therefore, the ERG’s preferred ICER per QALY gained remained at £82,743 per QALY gained, compared with the new ICER presented by the company (incorporating all of its changes) of £50,808 per QALY gained [13]. However, two sensitivity analyses were carried out by the ERG. First, the post-progression patient utility value was amended to the midway value between the ERG’s preferred estimate and that of the company’s. Second, the ERG applied K-M PFS data for the two treatment arms for the first 17 months, followed by a pooled extrapolation beyond 17 months. The rationale for this second sensitivity analysis was that a small (non-statistically significant) difference between treatment arms could be observed between 3 months and 17 months; this difference between treatment arms never exceeded 3 days at any point in time. The first sensitivity analysis reduced the estimated ICER by £12,900 to £69,843 per QALY gained and the second by £408 to £82,335 per QALY gained [13]. When both these sensitivity analyses changes were applied together, the ICER was £66,272 per QALY gained [13].

### Second Appraisal Committee Meeting

A number of observations were made by the AC at its second meeting. The key issues arising are summarised below.

#### Evidence for Clinical Effectiveness

The AC accepted that, despite some limitations, the subgroup data were the only appropriate evidence available to assess the effectiveness of eribulin compared with capecitabine. The AC considered that patients with disease that progresses after treatment with eribulin would be very likely to receive capecitabine on progression. It was noted that the company’s evidence suggested that this is not likely to result in improved OS when compared with capecitabine followed by any other active treatment (i.e. current clinical practice). The AC was not, therefore, persuaded that a clear benefit had been shown for offering eribulin second line compared with third line (third-line treatment being recommended in NICE’s guidance on eribulin after two or more previous chemotherapy regimens [10]).

#### Evidence for Cost Effectiveness

Although vinorelbine had been included as a possible comparator in the updated scope for the current appraisal [11], the AC was critical of employing it as a ‘blended’ comparator alongside capecitabine. First, no evidence for the efficacy of eribulin versus vinorelbine was presented by the company nor was there any evidence to support the assumption that capecitabine and vinorelbine had equal efficacy. Second, there was no evidence that vinorelbine would only be administered intravenously (rather than orally). Third, there was no evidence to support the assumption that there would be an equal split of capecitabine and intravenous vinorelbine used as second-line treatment options; on the contrary, the AC considered capecitabine would be preferred more often than vinorelbine. The AC also rejected the assumption that there was a PFS benefit for eribulin versus the ‘blended’ comparator (based on trial evidence of eribulin versus capecitabine in Study 301 [13, 14]) or that a 21 month treatment cap was a reasonable assumption. The AC concluded that, given the ongoing uncertainty about the most appropriate utility values to use, the ICER of £69,843 per QALY gained from the ERG’s first sensitivity analysis was the most plausible result [10].

## Conclusions

Eribulin appears to improve OS but not PFS for patients with HER2-negative LABC/MBC that has progressed after only one chemotherapy regimen. The reasons why this benefit is only experienced post progression in this group of patients is unclear.

The company and ERG disagreed on the most likely estimate of cost effectiveness. In most respects, the AC accepted the modifications to the company’s model made by the ERG, the exception being in relation to utility values, in which a mid-point estimate between that of the company and that of the ERG was preferred. This resulted in an ICER per QALY gained in excess of that usually considered by NICE to be cost effective, even in instances where NICE end-of-life criteria are applied. Therefore, NICE was unable to recommend eribulin for treating LABC/MBC in adults who have had only one chemotherapy regimen. However, eribulin remains a recommended treatment option for treating LABC/MBC in adults whose disease has progressed after at least two chemotherapy regimens.
